# Human gut flagellome profiling using FlaPro reveals TLR5-related phenotype-specific alterations in IBD

**DOI:** 10.1080/19490976.2026.2698917

**Published:** 2026-07-09

**Authors:** Anna A. Bogdanova, Andrea Borbón-García, Ruth E. Ley, Alexander V. Tyakht

**Affiliations:** a Department of Microbiome Science, Max Planck Institute for Biology Tübingen, Tübingen, Germany; b Cluster of Excellence EXC 2124 Controlling Microbes to Fight Infections, University of Tübingen, Tübingen, Germany

**Keywords:** Gut microbiota, machine learning, multi-omics integration, computational biology, IBD, silent flagellins, flagellome, pipeline, TLR5

## Abstract

Flagellin, the structural protein of bacterial flagella, activates the innate immune receptor Toll-like receptor 5 (TLR5). However, the ability of different flagellins to bind and stimulate TLR5 varies widely, suggesting that the composition of an individual's flagellin repertoire, defined as flagellome, may influence host–microbiome interactions and inflammation. Here, we developed FlaPro, a computational pipeline for quantification and functional annotation of human gut flagellomes. Functional categories in FlaPro are derived from a machine learning model trained on experimentally characterized flagellins with defined TLR5-binding and stimulatory activities. Application of FlaPro to a multi-omics inflammatory bowel disease (IBD) cohort revealed a marked depletion of flagellome diversity and a reduced ratio of silent to stimulatory flagellins in Crohn's disease and ulcerative colitis. These alterations were consistent across genomic and transcriptional layers, indicating a disease-associated shift toward more stimulatory flagellome profiles. Our findings suggest that specific features of the gut flagellome contribute to TLR5-mediated immune activation and may serve as functionally interpretable microbiome markers for future microbiome-wide association studies in health and disease. The workflow implemented in Snakemake is openly available at https://github.com/leylabmpi/FlaPro.

## Introduction

The gut microbiota plays an essential role in human physiology, contributing to digestion, interacting with the immune system and influencing distal organ systems. In the healthy state, the human gut microbiota is generally more commensal or beneficial than harmful; however, an imbalance (or dysbiosis) in microbial composition or gene expression activity can compromise host health. Dysbiosis, defined as a shift in microbiome composition away from that seen in healthy individuals, has been linked to a variety of disease conditions. A shift in gene expression patterns by the microbiome has also been observed in connection to disease. Shift in microbiome composition and/or activity can result in increased activation of innate immunity, leading to chronic inflammation.^1^

One mechanism by which the immune system recognizes bacteria is through pattern recognizing receptors (PRRs) such as Toll-like receptor 5 (TLR5), which detects flagellins and initiates immune response. Flagellin is the protein structural constituent of prokaryotic flagella, which act as locomotive nanomachines enabling bacterial movement. Due to its role in motility and surface exposure, flagellin serves as a key target for immune recognition. The structure of flagellins comprises two conserved domains, D0 and D1, at both the C-terminal and N-terminal ends, connected by a hypervariable region (HVR). Initial models based on the FliC flagellin of the pathogen *Salmonella* Typhimurium proposed the interaction between flagellin and human TLR5 involves three main steps: (1) TLR5 detects and binds to a conserved region in flagellins, triggering ligand-induced dimerization; (2) this leads to the recruitment of MyD88 and activates downstream cascades culminating in the activation of NF-κB; (3) NF-κB translocates to the nucleus and promotes transcription of pro-inflammatory cytokines.^2^ Subsequent studies have shown that *Helicobacter pylori* flagellins fail to bind and therefore evade TLR5-mediated detection.^3^ However, this prevailing model that flagellin-TLR5 binding led to activation failed to explain the poor agonism of flagellins produced by common gut commensal bacteria, such as the *Lachnospiraceae*.^4^

Flagellins with poor agonism elicit a TLR5 response at orders of magnitude higher concentrations than the canonical stimulatory flagellin FliC. Clasen et al. observed a so-called “silent” behavior, whereby flagellins bind TLR5 strongly at the D1 domain similarly to stimulatory flagellins, but fail to elicit a robust response. This poor agonism is due to the lack of a previously undescribed allosteric binding site on the D0 domain necessary for signalling.^4^ Based on these observations, flagellins tested for their TLR5 binding and activation could be classified into “stimulatory”, “evasive”, and “silent” classes.^4^

The human flagellome contains thousands of different sequence variants,^4^ or an order of magnitude more than have been profiled in the laboratory for their TLR5 binding and signalling. However, the information contained in the amino acid sequence alone is insufficient to predict with confidence the signalling class of a novel flagellin. The features that confer the TLR5 signalling properties of flagellin remain to be fully explored for the purpose of classifying novel flagellins into stimulatory, evasive, and silent categories. A model that could sort the flagellome into classes could inform the inflammatory potential of a microbiome without the necessity of arduous laboratory testing.

High-throughput metagenomics enables deep characterization of a human microbiome sample by capturing not just the taxonomic composition but also the functional potential. Metatranscriptomics extends this by revealing the actual expression of encoded functions. Associative studies of large cohorts have helped identify both shared and condition-specific microbial signatures of various human diseases. Given the immense dimensionality of such feature space—achieving the orders of hundreds of millions of features in gene catalogues^5^—practical analysis typically requires reducing it via feature aggregation or targeting specific functionally relevant gene classes (e.g. carbohydrate-active enzymes, antibiotic resistance genes and virulence factors, or pathway-based metabolic phenotypes).^6^ Although flagellins comprise a relatively compact family within the gut microbiome's gene repertoire, considering the variability of their TLR5 interaction phenotypes it is promising to: (1) characterize the complete pool of flagellins within individual samples (hereafter, the flagellome); and (2) explore associations of flagellome with host health, disease states or other factors.

Inflammatory bowel diseases (IBD)—ulcerative colitis (UC) and Crohn's disease (CD)—are marked by abnormal immune responses to commensal gut microbiome. Bacterial flagellins have been implicated in the onset and progression of these pathologies. There is a broad consensus that immune responses to flagellins play an important role in the CD pathogenesis. In particular, elevated levels of anti-flagellin antibodies (IgA and IgG) are consistently observed in CD patients and employed as diagnostic markers.^7–9^ These responses are typically mediated by flagellin-specific CD4⁺ T cells activated via TLR5 signalling pathways, driving the chronic intestinal inflammation. Of note, a *Lachnospiraceae* flagellin CBir1, the immunodominant antigen recognized by colitic mice and by approximately half of patients with CD,^10^ has been categorized as a “silent” flagellin.^4^ While the role of flagellin-specific immune responses in UC is less well defined, the presence of anti-flagellin antibodies in UC patients, although at lower prevalence,^11^ suggests a possible involvement in disease pathogenesis as well. Together, these observations suggest that silent flagellins may interact differently from stimulatory flagellins with adaptive immunity in IBD. However, to date, methods to sort flagellins from the flagellomes derived from metagenomes and metatranscriptomes are lacking.

Here, we developed FlaPro—a modular workflow for high-throughput quantification of the flagellome in human gut metagenomes and metatranscriptomes. Using experimentally derived data, we constructed a model predictive of TLR5 interaction phenotypes and a reference database of flagellins annotated with these predictions. We then performed a proof-of-principle flagellome-wide association study of IBD gut microbiome based on a published dataset.^12^

## Materials and methods

### Flagellin reference database

We obtained an initial human gut flagellin gene dataset (5,131 sequences) during our previous study^4^ by mapping a collection comprising over 33,000 flagellin protein sequences^13^ against human gut metagenomic data, with taxonomic classification for each flagellin obtained using the taxonomizR v0.10.6 R package.^14^ We curated the initial set by filtering out the sequences assigned to the taxa not previously reported in the human gut microbiota according to the GMrepo v2 database^15^ with the exception of opportunists or pathogens and transient species (i.e. food-associated species). This step resulted in the final dataset of 2,543 flagellin protein sequences.

### FlaPro model for predicting TLR5-related phenotypes

**Figure 1.**
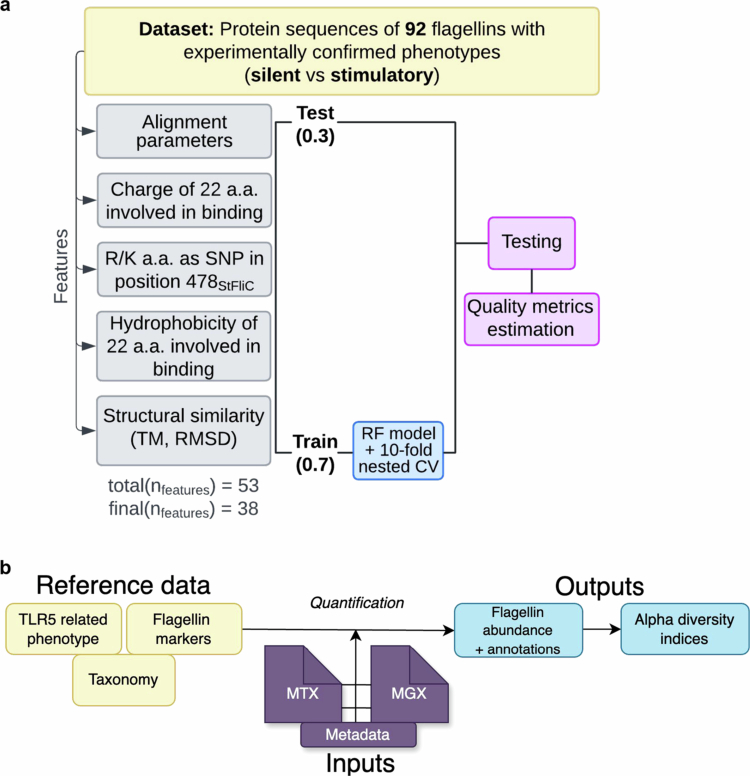
Overview of the FlaPro classification model and workflow. (a) Scheme of the classification model showing key components: sample size, phenotype labels, extracted features, and the machine learning algorithm. (b) Conceptual diagram of the FlaPro workflow. Using the curated reference data, the user-provided input (metagenomic or metatranscriptomic datasets, MGX or MTX) are processed through a modular pipeline.

To functionally annotate the flagellins as either “stimulatory” or “silent” with their respect to TLR5, we leveraged experimentally derived TLR5 binding and activation data available for 119 flagellins, using *St*FliC PIM (*Salmonella* Typhimurium FliC with mutations in primary interface) as a threshold for differentiating the classes.^4^ Since evasive flagellins were underrepresented in the experimental data (*n* = 27), we did not include them for the model training. As this annotated set represented only ~3.6% of the reference database, we trained a random forest model to predict TLR5-related phenotypes for the remaining sequences (Figure 1A). Model training employed 10-fold nested cross-validation on a stratified 70% training set with a held-out 30% test set reserved for final evaluation. The random forest was implemented using the randomForest R package v4.7-1.2 within the nestedcv v0.8.0 R framework,^16^ with mtry parameter as the sole tuned hyperparameter (optimal value: 2). A label permutation test with 100 label-shuffled iterations was conducted to assess the robustness of the model.

To predict TLR5 interaction phenotypes, we engineered 53 features grounded in experimental evidence of flagellin-TLR5 binding mechanisms that form four groups**:**

(i) Alignment-based features (*n* = 6): metrics (percent identity, alignment length, and mismatch count) produced by pairwise alignment of query flagellins to the C-terminal D1 and D0 domains of two reference flagellins: *Rh*FlaB (*Roseburia hominis* FlaB) as a representative silent flagellin and *St*FliC as a canonical stimulatory flagellin. The C-terminal D0 domain contains an additional allosteric binding site which is important for TLR5 signaling, as demonstrated before.^4^
(ii) Position-specific features (*n* = 1). This binary feature encoded whether a basic residue (arginine or lysine) is present at position 478 according to the *St*FliC sequence. Experimental mutagenesis demonstrated that this position modulates TLR5 activation.^4^ Query sequences were aligned to *St*FliC using MAFFT v7.520 to identify the corresponding position.
(iii) Physicochemical properties of TLR5-binding residues (*n* = 46 initial, *n* = 31 final). Twenty-two residue positions relevant for TLR5 recognition were identified from the resolved crystal structures of *Salmonella* Dublin (sdFliC) and *Bacillus subtilis* flagellins based on their crystal structures, structure-guided mutagenesis and functional studies primarily utilizing *St*FliC.^4,17–21^ Because *St*FliC and the crystallized sdFliC share virtually identical conserved D0/D1 domains, we used *St*FliC as the standardized reference. We derived the positions for the rest of sequences by mapping flagellins to *St*FliC using MAFFT v7.520.^22^ We used ordinal encodings for net charge (negative: −1, neutral: 0, positive: +1) and functional hydrophobicity (hydrophilic: R, N, D, Q, E, K; amphipathic: G, H, P, S, T, Y, W; hydrophobic: A, C, I, L, M, F, V, encoded as 1, 0, −1 respectively) based on Kyte-Doolittle scale thresholds.^23^
(iv) Structural similarity variables (*n* = 2). We included it in order to capture 3D structural variations to account for spatial conformations that 1D sequence alignments might miss. We quantified 3D structural similarity to *Rh*FlaB using Foldseek v10.941cd33: RMSD and TM-score. Protein structures for all flagellins were predicted using AlphaFold2.^^24.^^ Structural comparisons were restricted to the conserved D0 and D1 domains; hypervariable regions (HVR) were identified based on the conservation score and removed from the structures, as they are not essential for core TLR5 recognition and activation.^25^ ^,^ ^26^

Features with zero variance were removed prior to modeling resulting in the final list of 38 parameters.

The classification confidence score represents the signed distance from the decision boundary (*p* = 0.5) in probability space. Negative values indicate confidence toward stimulatory phenotype classification; positive values indicate confidence toward silent phenotype classification. Values near zero represent ambiguous cases where classification probability approaches the decision threshold. We used the score to assess the model decision confidence towards false predictions. The scoring formula was defined as:

$$S_{\mathrm{confidence}}=P(\mathrm{predicted}\;\mathrm{class})-0.5,$$

where the sign of the score indicates the direction of the predicted phenotype.

### Workflow for flagellome profiling

The FlaPro pipeline processes gut metagenomes to quantify the reads belonging to flagellins annotated as stimulatory or silent. We implemented the analysis using the Snakemake workflow management system.^27^ We organized the pipeline into modular components encompassing reference input handling and flagellin quantification. Input data consist of FASTQ or FASTA files derived from metagenomic (MGX) or metatranscriptomic (MTX) sequencing. In addition to user-provided microbiome datasets, the workflow requires three reference inputs: (1) the human gut flagellin marker sequence database, (2) taxonomical annotations, and (3) functional annotations of the flagellins. The pipeline processes these inputs through a set of modular steps, which can be selectively activated depending on the analysis objectives. Key steps include quantification of flagellins and assignment of taxonomical and functional labels (Figure 1B).

For quantifying the presence of specific flagellin sequences, we employed the ShortBRED tool v0.9.5, which consists of two main modules: ShortBRED Identify and ShortBRED Quantify.^28^ The Identify module clusters protein sequences based on homology and selects unique marker sequences for each cluster (used parameters: CD-HIT 0.95, marker length 30). These markers are subsequently used by Quantify to detect and quantify corresponding sequences in MGX/MTX datasets. From the curated set of 2543 flagellin sequences, ShortBRED generated 1322 clusters. Although cluster-level analysis reduces resolution, it confers a key advantage: flagellins with sequence variation that would otherwise evade exact database matching can still be detected within clusters, allowing broader coverage and improved sensitivity compared to traditional methods. We implemented a modification of the ShortBRED software by replacing USEARCH v11.0.667 with the DIAMOND2 v2.1.13 aligner, which is known for higher performance in large-scale analyses.^29^

The quantification module then uses the marker database to analyze MGX and MTX data. The output includes a table with columns for Flagellin ID, Counts, Hits, and TotalLengthMarker. When <50% of a cluster's marker sequences are matched by reads from a given sample, the abundance is deemed unreliable by ShortBRED and set to zero; otherwise, abundance is normalized to RPKM (reads per kilobase of reference sequence per million sample reads). To retain the discrete character of the data for downstream statistical analyses, we store original read counts—filtered by the 50% detection threshold—as “real counts”. In combination with taxonomic and predicted phenotype annotations, the resulting relative abundance matrix across multiple samples can be used to assess diversity (with alpha diversity integrated into the pipeline) and perform differential abundance analyses. The pipeline is available at: https://github.com/leylabmpi/FlaPro.

### IBD gut microbiome data

To explore the alterations of flagellome with disease, particularly in IBD, we applied FlaPro to a publicly available stool microbiome dataset from IBD patients and healthy subjects (*n* = 103) that included MGX and MTX data across multiple time points.^12^ After removal of one subject with missing age information, the cohort consisted of 26 healthy controls (HC), 28 individuals with ulcerative colitis (UC), and 50 with Crohn's disease (CD) (Supplementary Figure 1A).

### Associations between flagellome features and clinical status

Secondary analysis of the flagellome profiles was implemented as an R-based Jupyter notebook. To enhance reproducibility and facilitate code maintenance, we developed a modular system that assembles project notebooks from reusable, version-controlled code blocks, with reverse synchronization via Git. The analysis included two main approaches: component-based and compositional-aware, each offering distinct perspectives on flagellome variation.

In the component-based analysis, we focused on inter-individual variability in the proportion of flagellated bacteria—and thus flagellin abundance—in a sample. In accord, for both MGX and MTX data, relative abundance of each flagellin cluster in a sample was calculated by dividing the cluster's read counts by the total number of reads in the sample, scaled by a factor of 10^8^. Major flagellin clusters were defined as those with ≥30% prevalence across the samples. Leveraging paired MGX and MTX datasets of the IBD dataset,^12^ we quantified the transcriptional activity of each flagellin cluster by computing the MTX/MGX ratio—defined as the MTX-derived relative abundance divided by its MGX-derived counterpart (limited to the clusters with non-zero MGX abundance). For each of the three feature sets (MGX, MTX, and MTX/MGX ratio), we calculated per-sample flagellome alpha diversity indices (Chao1, Shannon, observed features) and the total flagellome relative abundance. These metrics were also stratified by flagellin class (stimulatory, silent, mixed, not defined); and the silent-to-stimulatory abundance ratio was computed. Pairwise dissimilarity of flagellome profiles was assessed using Euclidean distance. Differential abundance analysis for the IBD dataset was conducted on major flagellin clusters following log(x + 1) normalization, using a linear mixed-effects model (lmer function from the lme4 v1.1-37 R package), with clinical group, age and sample coverage (number of MTX or MGX reads) as fixed effects and subject ID as a random effect. Comparisons included both two-group tests (healthy controls vs. UC; healthy controls vs. CD) and a three-group test using a composite “disease score” variable (0 = HC, 1 = UC, 2 = CD) to reflect increasing disease severity. Multiple testing correction was applied using the Benjamini–Hochberg procedure, with a significance threshold of FDR < 0.05.

In the compositional-aware analysis (applied to both MGX and MTX data), flagellome profiles were treated as compositional data and analyzed using the Nearest Balance (NB, NearestBalance v0.2.0 R package). The NB method evaluates ratios of the features rather than their individual abundance values, and allows to identify two optimal subsets of features (flagellin clusters) whose normalized ratio (nearest balance) is associated with a variable of interest (such as disease) and can be computed as a single numerical value for any given sample.^30^

As required by the NB method, we first excluded samples with insufficient total flagellin abundance (<100 reads), then selected the most prevalent flagellin clusters using the same prevalence threshold as in the component-based approach, followed by a second filtering step retaining only clusters with total counts >30. General association between the factor (clinical status) and flagellome composition was tested using PERMANOVA (adonis2 function from vegan v2.6-10 R package; 9999 permutations). Identification of the nearest balance associated with clinical status was carried out as previously described,^31^ with flagellin clusters substituted for bacterial taxa. To account for repeated measurements across time points, each cross-validation iteration for nearest balance construction included a single randomly selected time point per subject (100 iterations). PERMANOVA was similarly applied across such iterations, and *p*-values from all runs were averaged to derive the final significance estimate.

### Data simulations for validation experiment

We simulated three metagenome-like datasets (average coverage = 135×) including paired-end Illumina reads using CAMISIM v1.3^32^ from all flagellin nucleotide sequences of the reference set (*n* = 2543). Their abundance profiles were based on the estimated distribution of flagellin presence in the human gut metagenomes.^4^ The profiles produced by FlaPro from these simulated metagenomes were compared to the expected ground truth at flagellin cluster level using multiple metrics including cluster retention rate, read retention rate, and correlation coefficients (Pearson and Spearman) between ground truth and FlaPro counts.

## Results

### Human gut microbiome derived flagellins predicted to be stimulatory or silent: FlaPro annotations and model

**Figure 2.**
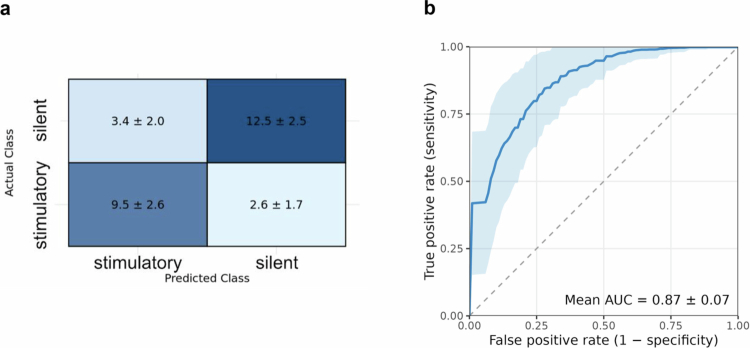
Performance evaluation of the classification model. (a) Confusion matrix averaged over 100 randomized iterations (mean ± standard deviation). (b) ROC curve averaged over 100 train/test splits (mean ± s.d. band).

Model performance was evaluated across 100 train/test splits. The resulting confusion matrix and AUC (area under the curve) (Figure 2) demonstrates reliable classification of flagellins into “stimulatory” and “silent” categories. Across 100 random train/test splits, the model classified flagellins with a mean accuracy of 0.79 ± 0.08—well above the no-information rate of 0.59—and a mean AUC of 0.87 ± 0.07. Per-class performance was balanced (sensitivity 0.76 ± 0.13, specificity 0.81 ± 0.11; balanced accuracy 0.79 ± 0.08). (Table 1). Random Forest feature importance analysis, assessed via mean variable importance across cross-validation folds (Supplementary Figure 2), revealed that structural similarity to *Rh*FlaB (RMSD via Foldseek) and sequence divergence from reference flagellins (mismatch count and percent identity to *St*FliC and *Rh*FlaB C-terminus domains) were the most stable and highest-ranked predictors. The binary feature encoding a basic residue at position 478 also ranked consistently high, in agreement with its experimentally demonstrated role in modulating TLR5 activation. Physicochemical properties at individual TLR5-binding positions contributed less individually but collectively captured variation across the binding interface. The consistently high performance across splits suggests the model effectively captures the key characteristics of the target phenotypes. Further validation through label randomization tests showed that predictive performance declined markedly when labels were randomized, indicating that the model learned genuine biological patterns rather than artifacts or biases (Supplementary Figure 3A and B).

**Table 1.** Model performance metrics summary.

Metric	Mean ± SD
Test accuracy	0.78 ± 0.08
Kappa	0.56 ± 0.16
Accuracy null	0.59 ± 0.06
Balanced accuracy	0.78 ± 0.08
Sensitivity	0.78 ± 0.13
Specificity	0.81 ± 0.11

Since we excluded evaders from the training dataset, the model does not explicitly predict the evasive phenotype. Instead, such sequences are classified into either the stimulatory or silent categories. When tested on the 27 known evasive flagellins, most were classified as silent (21 out of 27; 78%), which is biologically plausible given that both evaders and silent flagellins fail to elicit robust TLR5 activation, albeit through different mechanisms. Since most evasive flagellins will be classified as silent, and neither interact much with the receptor, broader interpretation of flagellome interactions with TLR5 will not be impacted.

Most of the cases that were misclassified in both training and test sets were located near the threshold separating the two phenotypes on the experimental TLR5 binding/activity plot (Supplementary Figure 4A), and in these cases, the predicted class probabilities were relatively low. Notably, the further a mismatch was from the experimental boundary, the lower the model's confidence in its prediction, indicating that misclassifications often reflect underlying biological ambiguity rather than model error.

The experimental classification threshold is based on binding and activity assay values for the “FliC PIM” mutant variant, which is impaired at the primary interface (D1) involved in TLR5 recognition.^4^ ^,^ ^18^ However, as previously reported,^4^ many flagellins display activity values close to this threshold. While strong binding at the primary interface can occur in both stimulatory and silent flagellins, signaling is believed to depend on an additional interaction at the D0 domain. This supports the idea that TLR5 interaction reflects a continuum rather than a binary trait—and suggests that the model captures this gradient to a meaningful extent.

Before applying the model to the full set of gut-derived flagellins, a small subset lacking key features was excluded, leaving 2462 sequences for prediction. Among these, the majority were classified as stimulatory, with silent flagellins comprising approximately 15% of the set (Supplementary Figure 4B). We also observed that some CD-HIT clusters used for ShortBRED marker construction contained flagellins with differing predicted phenotypes (Supplementary Figure 4C). To address this, we assigned a consensus phenotype to each cluster when one phenotype predominated and the classification confidence score for alternative types remained low (close to 0). The cases with low classification confidence across all members should be interpreted with caution.

### Validating FlaPro on simulated data

To evaluate the accuracy of FlaPro in detecting and quantifying diverse flagellin gene sequences in metagenomes, we tested the pipeline on a set of simulated metagenome sequences, which included the representative majority of flagellin gene sequences from the human gut derived flagellin database. FlaPro preserved flagellin community structure by retaining 99.7% of the flagellin clusters and their proportions, though the ranking of features is sometimes changed (Spearman *ρ* = 0.95; Supplementary Figure 5).

### Linking gut flagellome to disease: IBD

**Figure 3.**
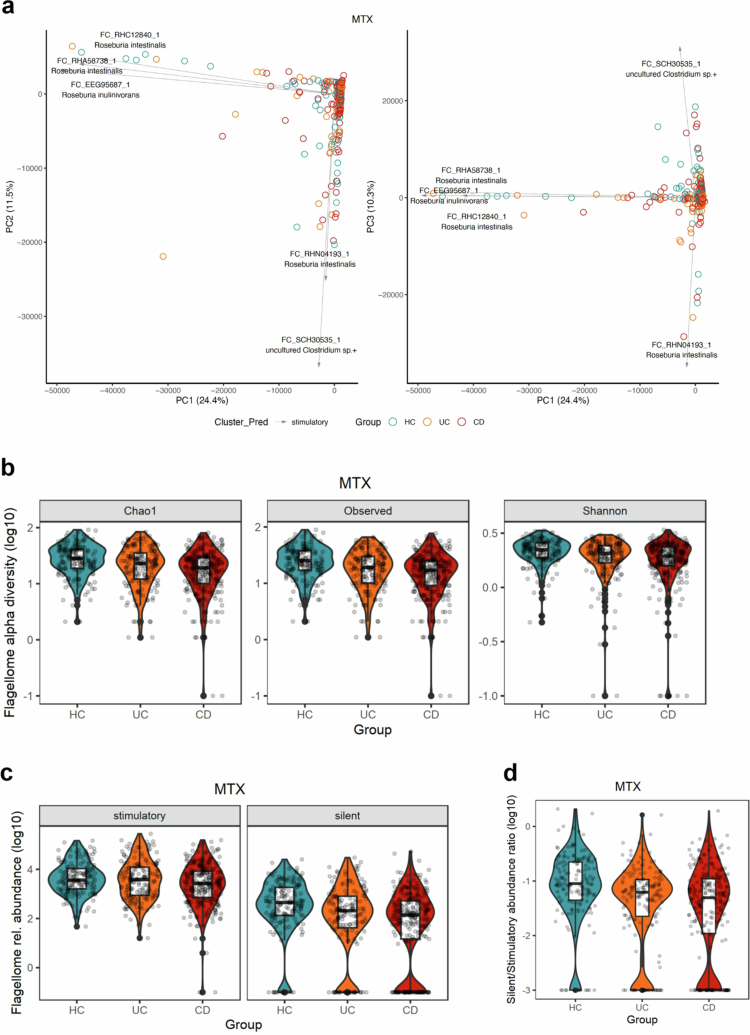
Altered gut flagellome expression in IBD. (a) Principal coordinate analysis (PCoA) of MTX-derived flagellome profiles. Biplots show the first three components, with arrows indicating the direction of the top five contributing features. Based on Euclidean distances between flagellin relative abundance profiles. Fourteen statistical outliers (>3 s.d. from the mean) were excluded for clarity (final *n* = 562 samples). (b) Reduced flagellome alpha diversity across IBD groups, measured by three indices. (c) Lower total flagellin relative abundance in IBD. (d) Decreased silent-to-stimulatory flagellin expression ratio. Each point represents one sample (multiple time points per subject are presented); distributions are shown as violin plots with log-scaled y-axes. *p*-values are not displayed on the plots, as the repeated-measures design precludes the use of standard independence-assuming tests; significance was instead assessed via linear mixed-effects models (see Supplementary Tables 1 and 2 and Methods). For (b and c), a pseudocount of 0.1 had been added to the values prior to plotting; for (d) −0.001.

Processing of a published microbiome dataset from IBD patients and healthy subjects^12^ in FlaPro showed that, among the 1322 flagellin clusters in the reference database, only 22 (MGX) and 14 (MTX) were prevalent under our defined threshold (SupplementaryFigure 1B and C; see Methods). In line with our earlier analysis,^4^ experimentally tested flagellins comprised a small fraction of the detected flagellome (Supplementary Figure 6A). Silent flagellins were consistently less abundant than stimulatory ones—by approximately an order of magnitude—according to both experimental and predicted annotations (Supplementary Figure 6B), with the stimulatory group being the primary contributors to the inter-sample variation (e.g., MTX: Figure 3A).

When applying alpha diversity metrics to the data, we observed IBD was associated with a reduced flagellome repertoire—at the level of encoded potential (MGX) as well as of the expression (MTX) (Figure 3B). This reduction was more pronounced in CD than in UC, particularly when comparing all three groups using the Chao1 metric in MTX (*p* = 4 × 10^−4^, linear mixed-effects model; see Methods). Stratified analysis showed this diversity loss affected both silent and stimulatory flagellins (Supplementary Figure 7; Supplemental Tables 1 and 2). However, it was only for the silent flagellins that we observed their total abundance reduced in both UC and CD compared to HC (for MTX – Figure 3C – and for MTX/MGX; Supplemental Tables 3–5)—in line with the decreasing silent:stimulatory abundance ratio (MGX and MTX; Figure 3D). Notably, the clinical metadata—as is characteristic of IBD cohorts—comprised numerous heterogeneous variables, often categorical and incomplete. This complexity precluded robust integration of all covariates into a single unified model without a significant loss of statistical power. Consequently, we conducted a targeted post-hoc visual analysis of key factors – disease activity and anatomical location. This assessment demonstrated that the metatranscriptomic (MTX) silent:stimulatory flagellin ratio—a major observation—did not exhibit substantial variation across these clinical strata (Supplementary Figure 8). Records of concurrent medications exhibited high dimensionality and longitudinal variability, rendering formal analysis unfeasible; consequently, they were excluded from the analysis.

**Figure 4.**
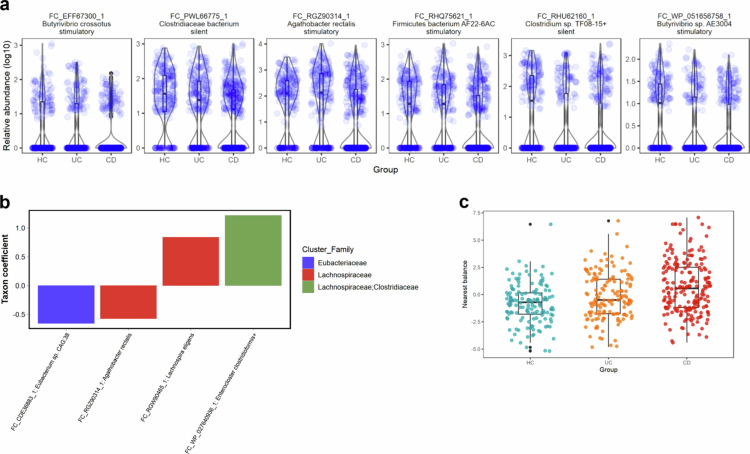
Differential analysis of flagellome-encoding potential in metagenomes. (a) Component-based analysis across the clinical groups using a “disease score” variable (linear mixed-effects model on log-transformed data; adjusted *p* < 0.05). Compositional-aware analysis (CD vs. HC): (b) Flagellin clusters with positive coefficients (numerator of the nearest balance) are enriched in CD as compared to HC, while those with the negative ones (denominator) are depleted. (c) Balance values calculated for each metagenome using the centered log-ratio (clr)-transformed values, adjusted for age, sample coverage and sex; values for the UC subjects were calculated using the CD-vs-HC balance and are provided for reference.

Pathology-associated flagellome depletion was also evident at the individual cluster level. Differential analysis using the DiseaseScore variable in a three-group comparison showed that out of 22 major flagellin clusters, six (four stimulatory and two silent) were significantly decreased in abundance in MGX (adj. *p* < 0.05; Figure 4A); at the level of expression, the respective number was four and for the MTX/MGX ratio—two (Supplementary Tables 6–8). More specifically, in CD patients relative to healthy controls, we observed reduced metagenomic abundance of flagellin clusters, primarily from commensal genera such as *Butyrivibrio* and *Agathobacter* (Supplementary Figure 9, Supplementary Table 9). MTX analysis showed decreased expression for three clusters, while the MTX/MGX ratio revealed reductions in two (Supplementary Figure 9; Supplementary Tables 10 and 11), suggesting the pathology-associated depletion of expressed flagellome occurs not just due to the decreased representation of the respective flagellated bacteria in the community but also due to the transcriptional downregulation within the respective taxa. In UC, although some associations yielded low raw *p*-values, none remained significant after correction for multiple testing—likely reflecting the smaller group size and, potentially, the milder clinical phenotype compared to CD.

From the alternative—compositional-aware—perspective, there was an overall tendency of link between the flagellome and IBD via MGX (DiseaseScore across all three groups: *p* = 0.06 ± 0.14, median ± s.d., repetitive-measures-aware PERMANOVA, see Methods), but not via MTX (median *p* > 0.2); this effect for MGX appeared driven primarily by CD rather than UC (Supplementary Table 12). For the CD vs. HC comparison in MGX, the nearest balance consisted of two flagellin clusters positively associated with the disease (numerator) and two negatively associated (denominator) (Figure 4B and C). We did not observe a clear enrichment of either silent or stimulatory classes in either part of the balance. Comparison of the component-based and compositional scenarios' results suggests that the altered ratios between the distinct flagellins are less likely to play a role in IBD than their abundance relative to all genes of the bacterial community.

## Discussion and conclusions

Flagellin diversity is vast and this diversity has functional consequences with respect to host immune receptors. For the human innate immune receptor TLR5, flagellins can range how they tune the receptor response from highly stimulatory to silent. Here, we developed FlaPro to predict the TLR5 response of a flagellin from its sequence and other features. Cross-validation results suggest considerable performance of the prediction. We then integrated FlaPro into a workflow allowing the user to input MGX and MTX data for flagellin quantification, classification, and to understand the underlying dynamics of differences at the level of microbiome diversity versus expression patterns.

We demonstrated the utility of our FlaPro pipeline for profiling the flagellome and gaining insights into host–microbiome interactions using a publicly available multi-omics IBD gut microbiome dataset.^12^ Acknowledging that clinical and pharmacological heterogeneity remain significant confounders in IBD cohorts and the observed changes in flagellin expression could reflect in particular altered intestinal transit kinetics and nutrient availability in the inflamed gut, we used this relatively small dataset as a preliminary investigation demonstrating the ability of the tool to detect and quantify differences in the flagellomes of different patient types and controls. Going forward, longitudinal analysis of patients and controls, much larger sample sizes – preferably balanced with respect to clinical variables like disease activity, location and medications – or highly controlled animal experiments could provide sufficient power to start relating specific flagellin changes to inflammatory or specific immune responses.

Given these caveats, the alterations of the flagellome observed in this IBD dataset may provide some initial insight into how specific bacterial components may contribute to disrupted immune response to commensal microbiome. As FlaPro is primarily a hypothesis-generating tool, we outline potential mechanisms behind the reduced silent:stimulatory ratio. First, the ratio shift along with the diversity reduction reflects the depletion of health-associated *Lachnospiraceae*, which contains both stimulatory and the majority of known silent flagellins, and bloom of opportunistic *Proteobacteria* with predominantly highly stimulatory ones. Second, transcriptional downregulation of flagellin genes within remaining taxa may further contribute beyond composition shifts alone. Investigation of larger sample sizes with consideration of IBD clinical subtypes and severity, particularly in treatment-naive patients, will provide a more detailed understanding of how the flagellin-encoding potential and expression vary in these diseases as well as future *in vivo* studies are needed to validate these FlaPro-derived functional insights.

The downstream secondary data analysis with an R notebook generator functionality can be flexibly adjusted to most common study design schemes. The raw features produced by the primary analysis can be readily input to feature engineering routines (for example, co-occurrence-based clustering) and more elaborate statistical approaches to differential abundance analysis (including those accounting for sparsity in data).

Our annotation strategy combining structure- and sequence-based prediction of function is extendable beyond human TLR5 to investigate flagellin interactions in other model animals (such as mice with their divergent TLR5 structure), in diverse niches (in environmental microbiome or plant microbiome). More broadly, this approach can be generalized to study other bacterial protein families that interact with host receptors, facilitating comprehensive, receptor-specific analyses of host–microbiome dynamics across a range of biological contexts.

Our methodology is not free from limitations. Firstly, a few of them are related to its relatively small set of the experimentally evaluated flagellins, which was constructed with a bias towards healthy subjects' microbiome and has underrepresented evasive phenotype due to the lack of known cases to date. When interpreting the silent-to-stimulatory flagellin ratio, it is important to consider that most of the true evaders fall into our “silent” category. Future work with expanded training datasets including more evasive flagellins will enable three-class prediction to address this limitation directly. A notable aspect of the feature space is its reliance on sequence and structural similarity to two reference flagellins, which dominate the feature importance ranking (Supplementary Figure 2). This reflects the current state of knowledge: these two flagellins represent the best-characterized examples of their respective phenotypes and anchor opposite ends of the TLR5 activation spectrum. Structural features depend on the AlphaFold2 predictions, which may not fully reflect natural structural properties of all flagellins in the dataset. Furthermore, our sequence-based features and experimental data do not account for native post-translational modifications. Consequently, our model predicts the baseline TLR5 interaction potential of the unmodified protein backbone.

In addition, in the current implementation of the feature construction, some flagellin clusters turn out to be mixed (by combining silent and stimulatory flagellins) thus complicating further interpretation of these features. In the IBD dataset analyzed here, the mixed clusters were low abundant and not associated with the clinical status. However, if profiling of future datasets produces associations for such features, their TLR5-related phenotypes can be elucidated experimentally. Additionally, as flagellins constitute a single protein with often very similar amino acid sequences, there is a risk of false positive assignments, especially when analyzing short-read sequences. Pipeline validation relied on simulated metagenomes, which is an unavoidable limitation given the absence of ground-truth experimental metagenomes with known flagellin abundances; furthermore, these simulations were constructed exclusively from flagellin sequences, assessing quantification accuracy rather than specificity against non-flagellin backgrounds. False positive assignments from non-flagellin reads are nonetheless mitigated by the ShortBRED marker selection step, which retains only peptide sequences unique to each flagellin cluster and absent from broader protein databases. Once a sufficient volume of long-read microbiome sequencing datasets becomes available, our pipeline can be updated to enable processing of the long reads to increase the profiling accuracy.

We anticipate that profiling of metagenomes from patients with diverse immune-related disorders using FlaPro will shed more light onto the contribution of gut microbiome to their pathogenesis. Future profiling of large metagenomic and metatranscriptomic datasets using FlaPro will help define the landscape of flagellome in health and uncover both condition-specific and shared alterations across diseases.

## Supplementary Material

Supplemental Material Supplementary_Figures_caption_07_Jul_2026_09_55_AU.docx

Suppl_Figures.pdf Supplemental Material

Supplemental_Tables.xlsx Supplemental_Tables.xlsx

## Data Availability

FlaPro and reproducible analysis scripts used in this study are available at https://github.com/leylabmpi/FlaPro.
