# Supplementary material for: Human gut flagellome profiling using FlaPro reveals TLR5-related phenotype-specific alterations in IBD
Source: Gut Microbes. 2026 Jul 9;18(1):2698917. doi: 10.1080/19490976.2026.2698917 (PMC13353774; doi:10.1080/19490976.2026.2698917)
Supplement: Supplemental Material — Supplementary_Figures_caption_07_Jul_2026_09_55_AU.docx [file KGMI_A_2698917_SM4705.docx]

**SFigure 1 - General information on IBD flagellome dataset.** (A) Distribution of available time points per subject. (B) Number of reads mapped to the flagellin reference set, separated by MGX and MTX layers. (C) Prevalent flagellin clusters (prevalence >30%) with their predicted phenotypes.

**SFigure 2 - Feature importance in the classification model.** Mean variable importance across model folds; error bars indicate standard error of the mean (SEM). Color indicates direction of association (stimulatory vs. silent). Asterisks (*) mark high-impact sequence alignment-based features.

**SFigure 3 - Label shuffling test across 100 iterations.** (A) Confusion matrices for correct (left) and shuffled (right) labels; mean values ± SEM. (B) Significance from a Benjamini–Hochberg-corrected label-permutation test (* p_adj < 0.05);

**SFigure 4 - Predicted flagellin phenotypes.** (A) TLR5 binding/activation plot based on Clasen et al., 2023 data with experimentally defined phenotypes shown as color-filled background regions and predicted phenotypes’ classification confidence score overlaid as gradient-colored points (red: stimulatory, blue: silent). Dots with a black border reflect low-confidence classification (classification confidence score = 0). (B) Proportions of predicted silent, stimulatory, and unclassified flagellins in the gut microbiome database. (C) Distribution of mixed phenotype clusters (as used by ShortBRED *quantify* module). Top: number of clusters with conflicting phenotype assignments. Bottom: prediction probabilities indicating confidence of phenotype assignment for mixed clusters.

**SFigure 5 - Validation on simulated data.** (A) Pipeline validation summary. Colors code the category of the validation criteria; the black dashed line indicates 80%. (B) Relative abundance comparison. The green dashed line indicates perfect correlation. Each dot is a flagellin cluster.

**SFigure 6 - The most abundant flagellin clusters in the IBDMDB dataset.** Ranking of the top 200 clusters by mean relative abundance in MTX data. (A) Annotated based on experimental data. (B) Annotated according to the predicted phenotype.

**SFigure 7 - Flagellome alpha diversity stratified by flagellin class.** (A) MGX data. (B) MTX data. Diversity metrics stratified by flagellin phenotype (stimulatory, silent, mixed, undefined).

**SFigure 8 - Exploring the links of flagellome expression with additional clinical variables in the IBD cohort.** Metatranscriptomic data: silent:stimulatory ratio of repeated measures per subject plotted against baseline disease activity score and disease location: (A,B) - CD and (C,D) - UC, respectively. In (A) and (C), point color encodes participant ID.

**SFigure 9 - Differential abundance of flagellin clusters in CD vs. HC.** Component-based analysis across the prevalent flagellin clusters for: (A) MGX, (B) MTX, and (C) MTX/MGX ratio. Linear mixed-effects model applied to log-transformed values (adjusted p < 0.05). "+" indicates clusters with multiple taxa (only the first taxon shown; the full list in the Supplemental Tables 9-11). Point color indicates direction of change (blue = decreased in CD; red = increased).
