# Supplementary material for: Human gut flagellome profiling using FlaPro reveals TLR5-related phenotype-specific alterations in IBD
Source: Gut Microbes. 2026 Jul 9;18(1):2698917. doi: 10.1080/19490976.2026.2698917 (PMC13353774; doi:10.1080/19490976.2026.2698917)

**SFigure 1**

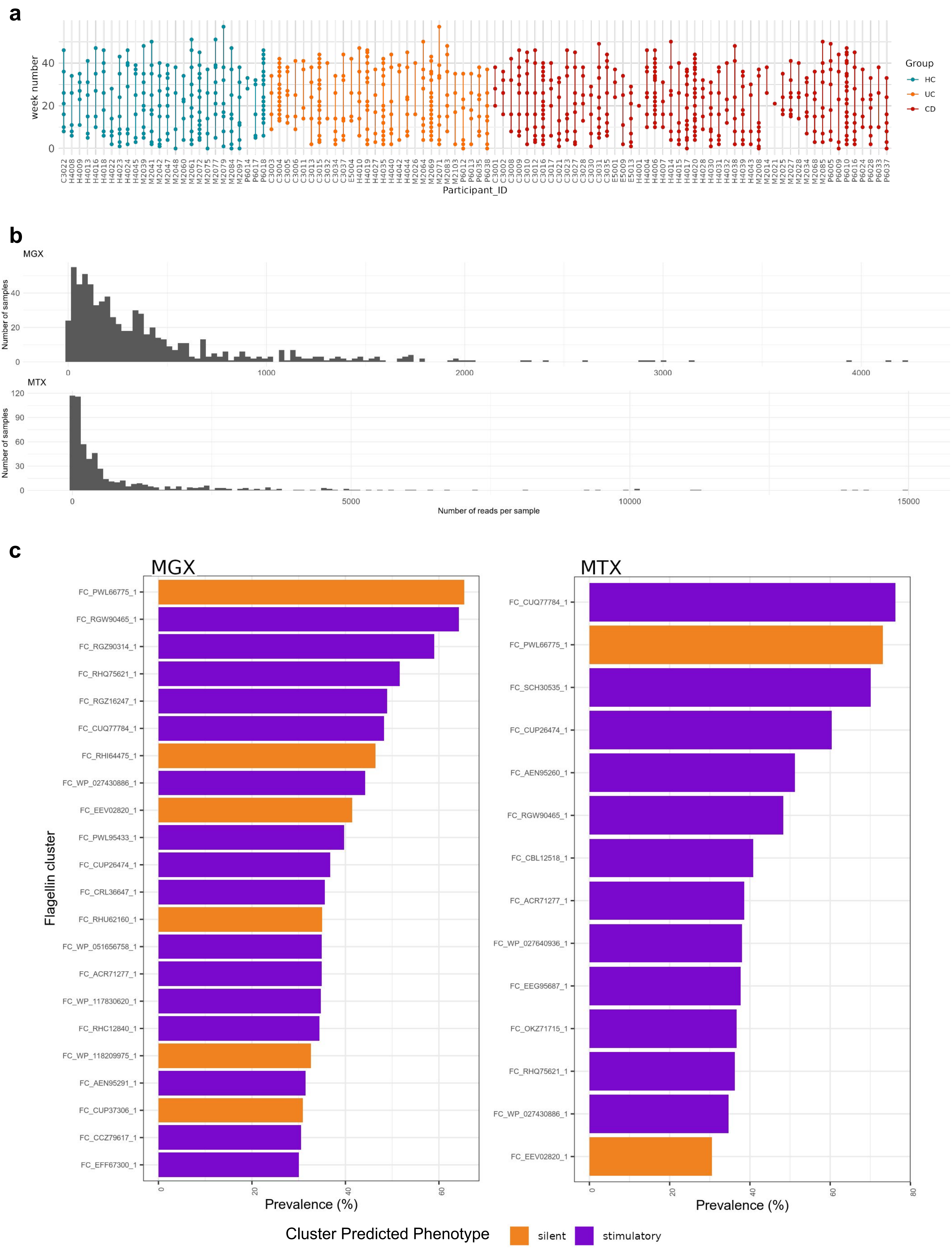
a
week number
0
20
40
Participant_ID
C3022 H4008 H4009 H4013 H4016 H4018 H4022 H4023 H4024 H4045 M2039 M2041 M2042 M2047 M2048 M2060 M2061 M2072 M2075 M2077 M2079 M2084 M2097 P6014 P6017 P6018 C3003 C3004 C3005 C3006 C3011 C3013 C3015 C3032 C3034 C3037 E5004 H4010 H4019 H4027 H4035 H4040 H4042 H4044 M2026 M2064 M2069 M2071 M2083 M2103 P6012 P6013 P6035 P6038 C3001 C3002 C3008 C3009 C3010 C3012 C3016 C3017 C3021 C3023 C3027 C3028 C3030 C3031 C3035 E5001 E5009 E5013 H4001 H4004 H4006 H4007 H4014 H4015 H4017 H4020 H4028 H4030 H4031 H4032 H4038 H4039 H4043 M2008 M2014 M2021 M2025 M2027 M2028 M2034 M2068 M2085 P6005 P6009 P6010 P6016 P6024 P6028 P6033 P6037
Group
HC
UC
CD
b
MGX
Number of samples
0
20
40
0
1000
2000
3000
4000
MTX
Number of samples
0
30
60
90
120
0
5000
10000
15000
Number of reads per sample
c
MGX
Flagellin cluster
FC_PWL66775_1
FC_RGW90465_1
FC_RGZ90314_1
FC_RHQ75621_1
FC_RGZ16247_1
FC_CUQ77784_1
FC_RHI64475_1
FC_WP_027430886_1
FC_EEV02820_1
FC_PWL95433_1
FC_CUP26474_1
FC_CRL36647_1
FC_RHU62160_1
FC_WP_051656758_1
FC_ACR71277_1
FC_WP_117830620_1
FC_RHC12840_1
FC_WP_118209975_1
FC_AEN95291_1
FC_CUP37306_1
FC_CCZ79617_1
FC_EFF67300_1
0
20
40
60
Prevalence (%)
MTX
FC_CUQ77784_1
FC_PWL66775_1
FC_SCH30535_1
FC_CUP26474_1
FC_AEN95260_1
FC_RGW90465_1
FC_CBL12518_1
FC_ACR71277_1
FC_WP_027640936_1
FC_EEG95687_1
FC_OKZ71715_1
FC_RHQ75621_1
FC_WP_027430886_1
FC_EEV02820_1
0
20
40
60
80
Prevalence (%)
Cluster Predicted Phenotype
silent
stimulatory

**SFigure 2**

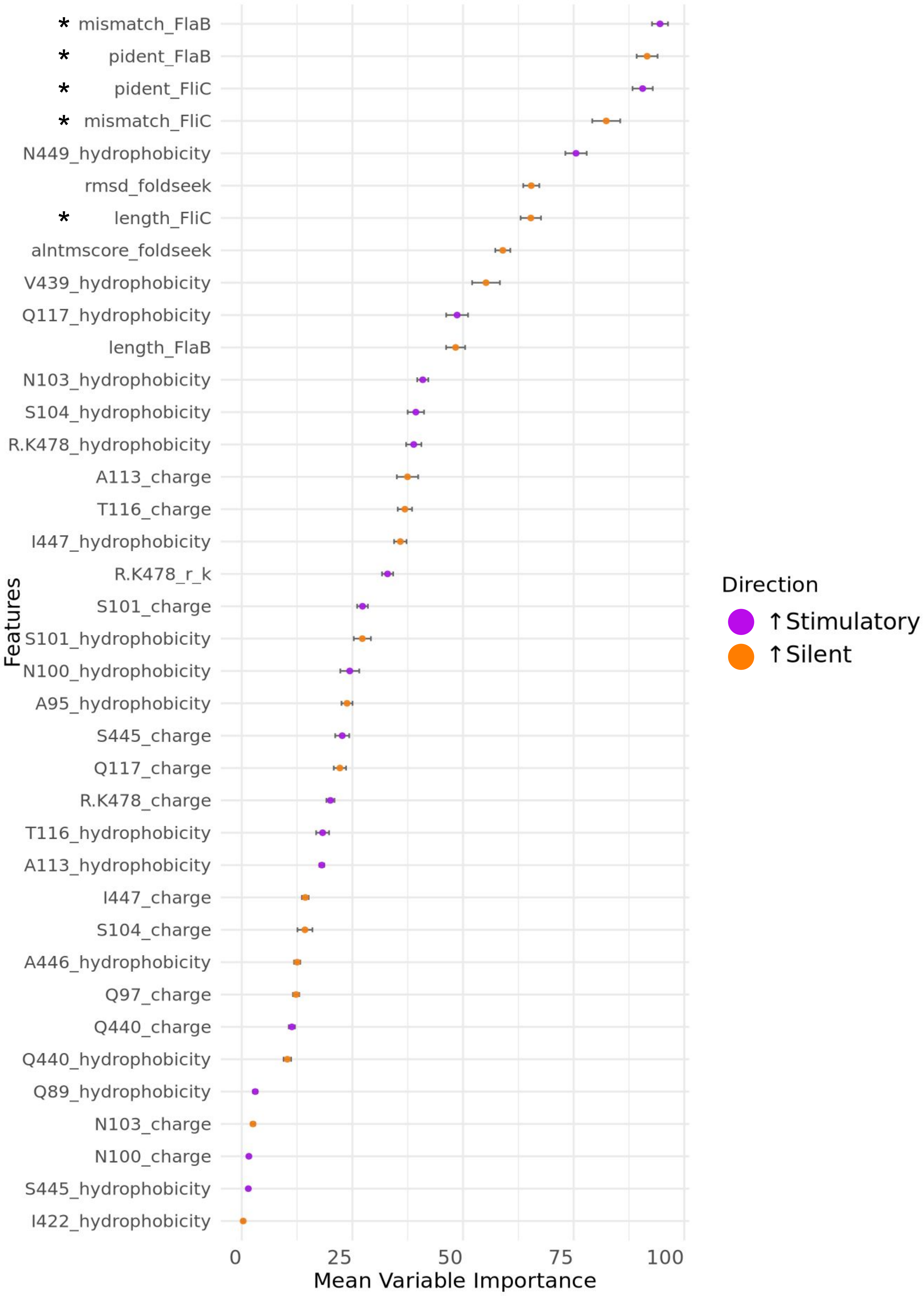
* mismatch_FlaB
* pident_FlaB
* pident_FliC
* mismatch_FliC
N449_hydrophobicity
rmsd_foldseek
* length_FliC
alntmscore_foldseek
V439_hydrophobicity
Q117_hydrophobicity
length_FlaB
N103_hydrophobicity
S104_hydrophobicity
R.K478_hydrophobicity
A113_charge
T116_charge
I447_hydrophobicity
R.K478_r_k
S101_charge
S101_hydrophobicity
N100_hydrophobicity
A95_hydrophobicity
S445_charge
Q117_charge
R.K478_charge
T116_hydrophobicity
A113_hydrophobicity
I447_charge
S104_charge
A446_hydrophobicity
Q97_charge
Q440_charge
Q440_hydrophobicity
Q89_hydrophobicity
N103_charge
N100_charge
S445_hydrophobicity
I422_hydrophobicity
Features
0
25
50
75
100
Mean Variable Importance
Direction
↑Stimulatory
↑Silent

**SFigure 3**

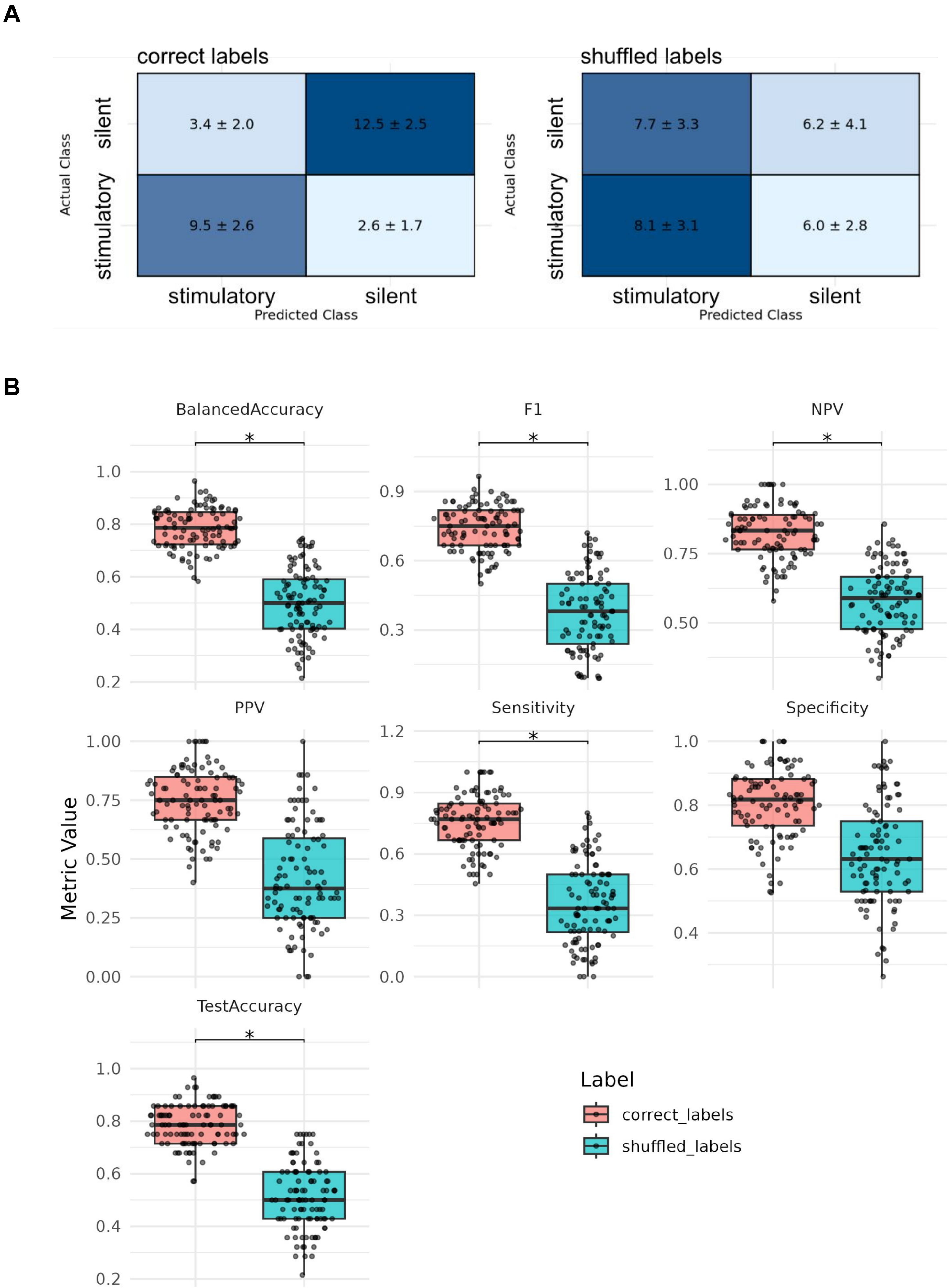
A
correct labels
Actual Class
silent
stimulatory
3.4 ± 2.0
12.5 ± 2.5
9.5 ± 2.6
2.6 ± 1.7
stimulatory
silent
Predicted Class
shuffled labels
Actual Class
silent
stimulatory
7.7 ± 3.3
6.2 ± 4.1
8.1 ± 3.1
6.0 ± 2.8
stimulatory
silent
Predicted Class
B
BalancedAccuracy
F1
NPV
PPV
Sensitivity
Specificity
TestAccuracy
*
Metric Value
Label
correct_labels
shuffled_labels

**SFigure 4**

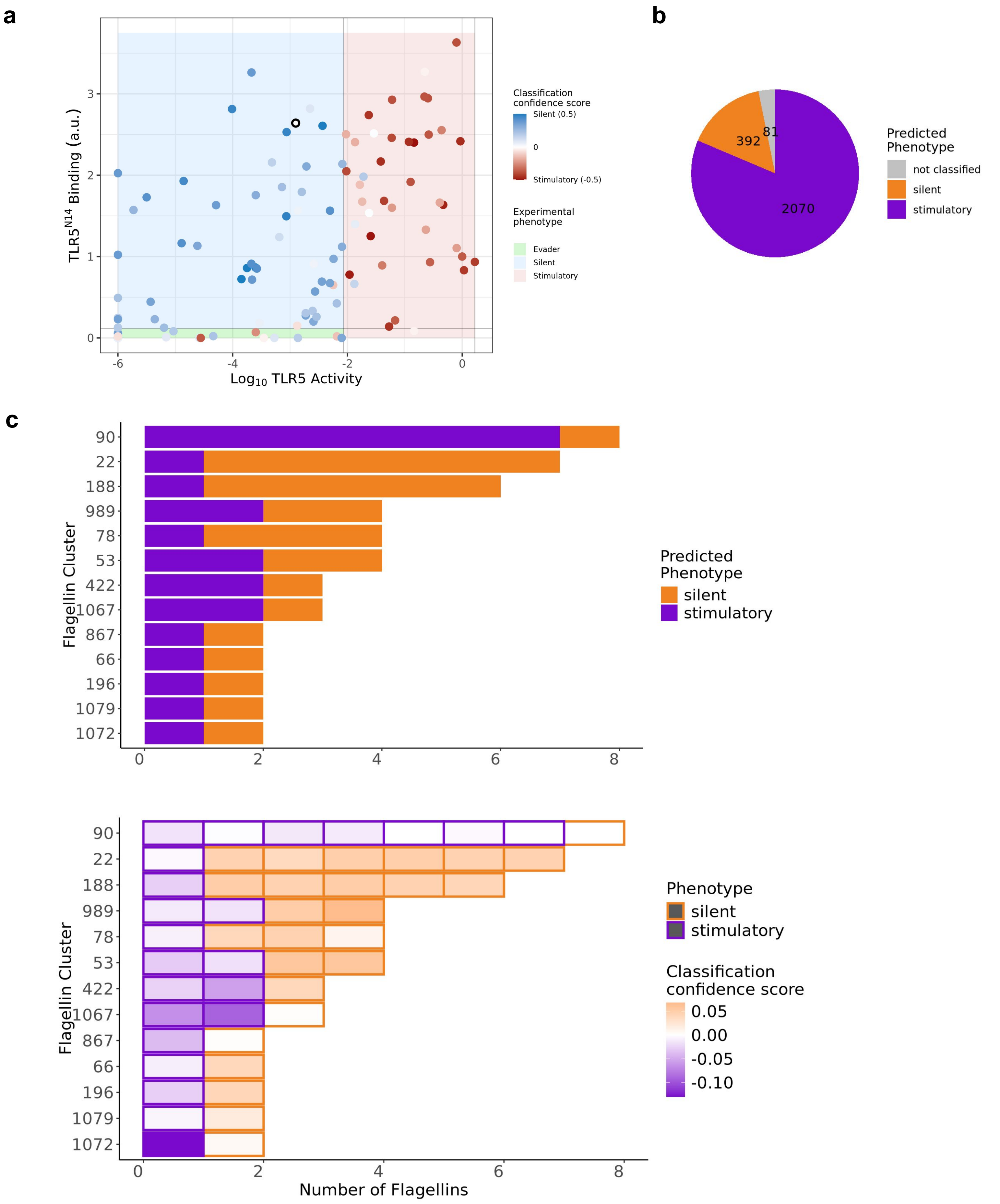
a
TLR5$^{N14}$ Binding (a.u.)
Log$_{10}$ TLR5 Activity
Classification confidence score
Silent (0.5)
0
Stimulatory (-0.5)
Experimental phenotype
Evader
Silent
Stimulatory
b
392
81
2070
Predicted Phenotype
not classified
silent
stimulatory
c
Flagellin Cluster
90
22
188
989
78
53
422
1067
867
66
196
1079
1072
Predicted Phenotype
silent
stimulatory
Number of Flagellins
Phenotype
silent
stimulatory
Classification confidence score
0.05
0.00
-0.05
-0.10

**SFigure 5**

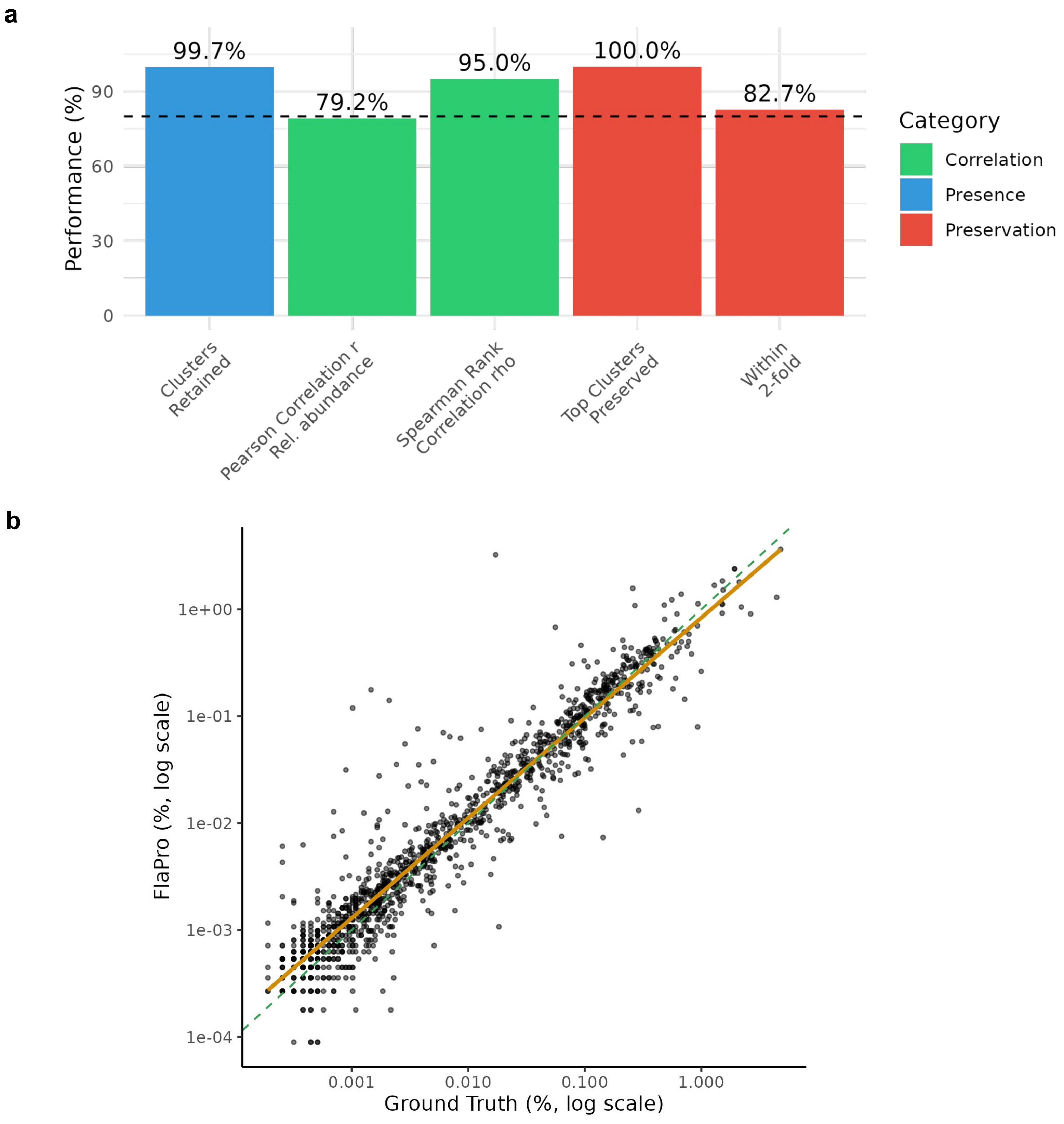
a
99.7%
79.2%
95.0%
100.0%
82.7%
Performance (%)
0
30
60
90
Clusters Retained
Pearson Correlation r Rel. abundance
Spearman Rank Correlation rho
Top Clusters Preserved
Within 2-fold
Category
Correlation
Presence
Preservation
b
FlaPro (%, log scale)
1e+00
1e-01
1e-02
1e-03
1e-04
0.001
0.010
0.100
1.000
Ground Truth (%, log scale)

**SFigure 6a**

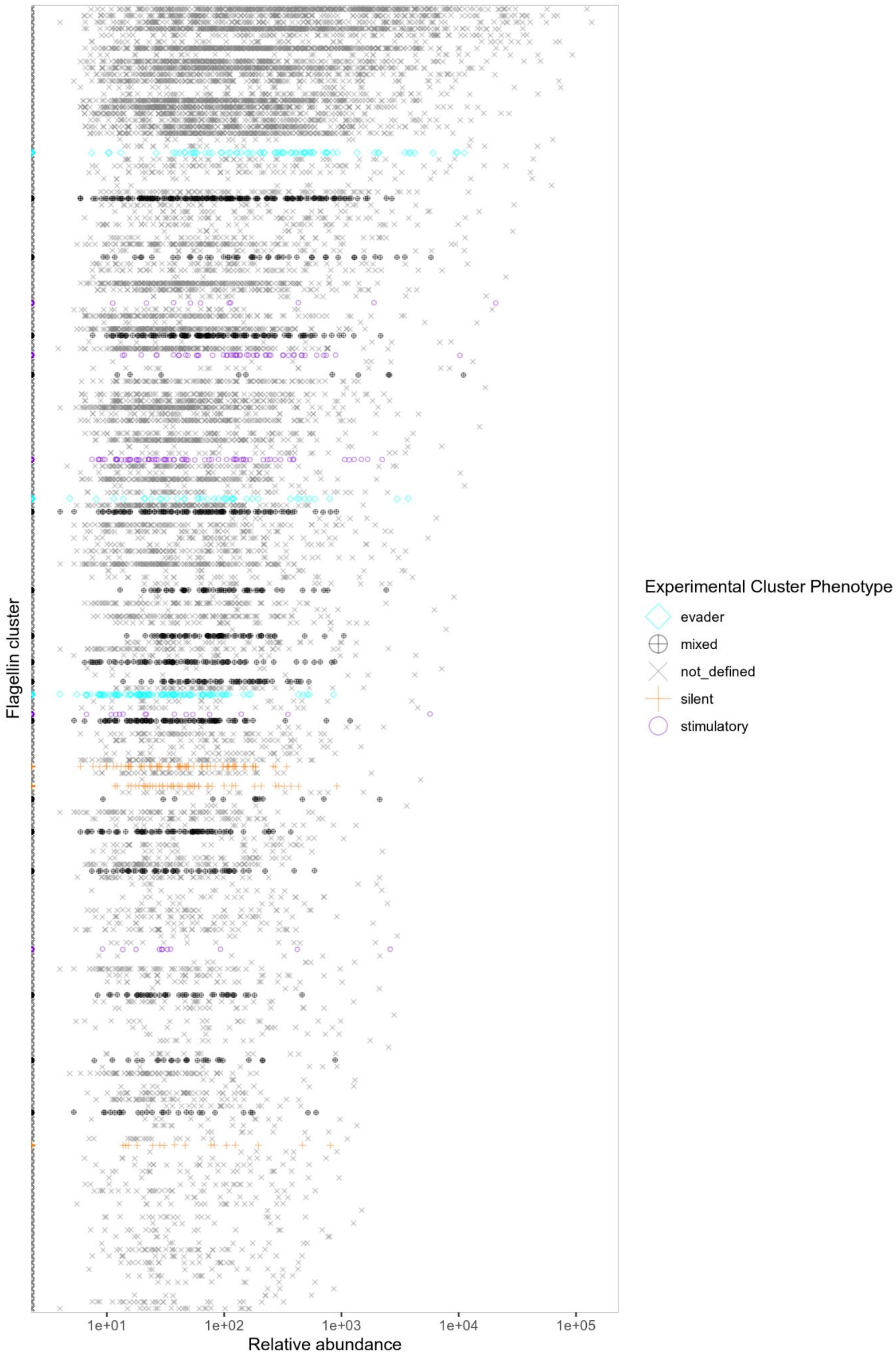

Flagellin cluster
1e+01
1e+02
1e+03
1e+04
1e+05
Relative abundance
Experimental Cluster Phenotype
evader
mixed
not_defined
silent
stimulatory

**SFigure 6b**

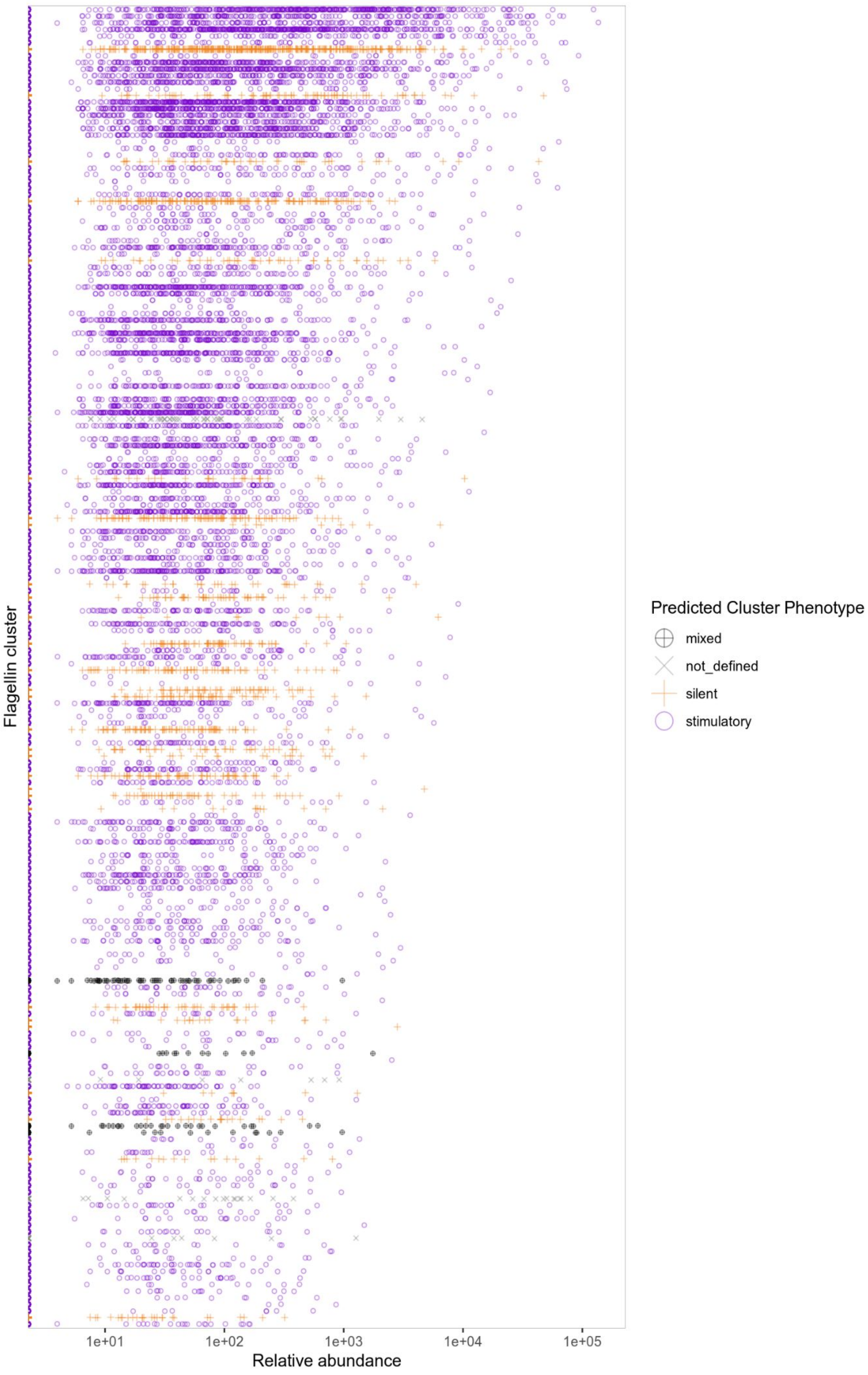

Flagellin cluster
1e+01
1e+02
1e+03
1e+04
1e+05
Relative abundance
Predicted Cluster Phenotype
mixed
not_defined
silent
stimulatory

**SFigure 7**

**a**

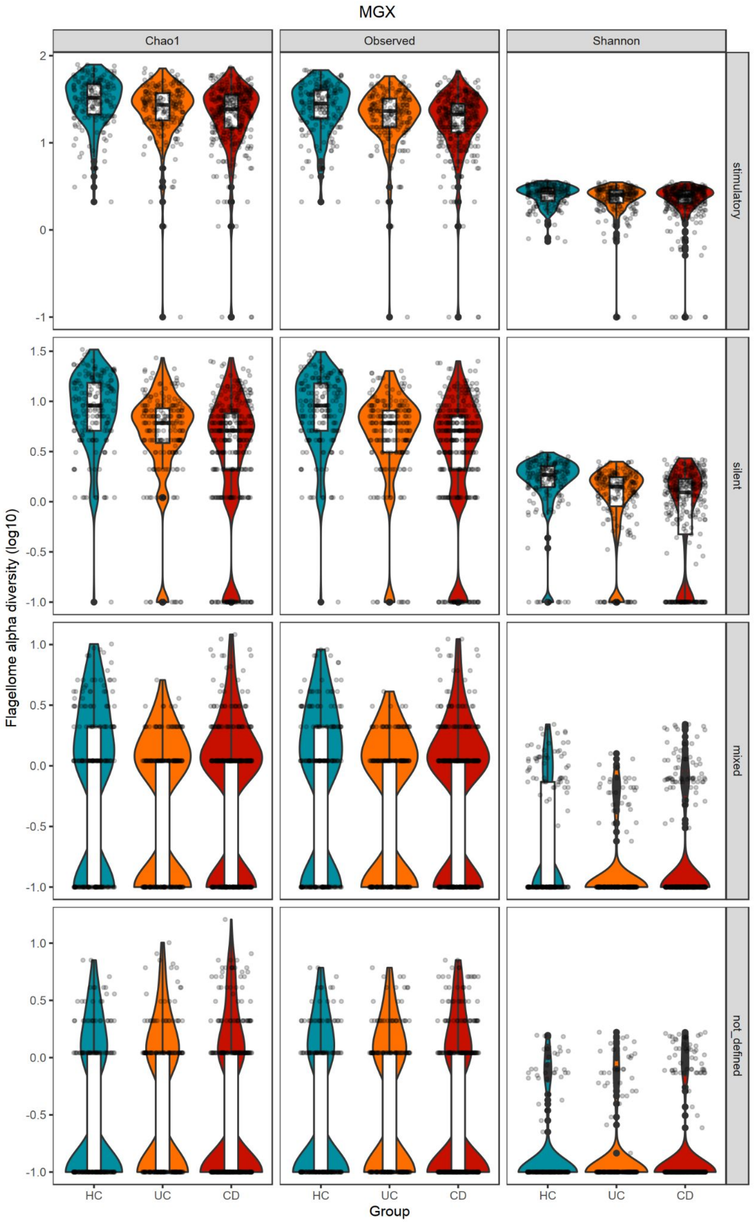

MGX
Chao1
Observed
Shannon
stimulatory
silent
mixed
not_defined
Flagellome alpha diversity (log10)
HC
UC
CD
Group


**b**

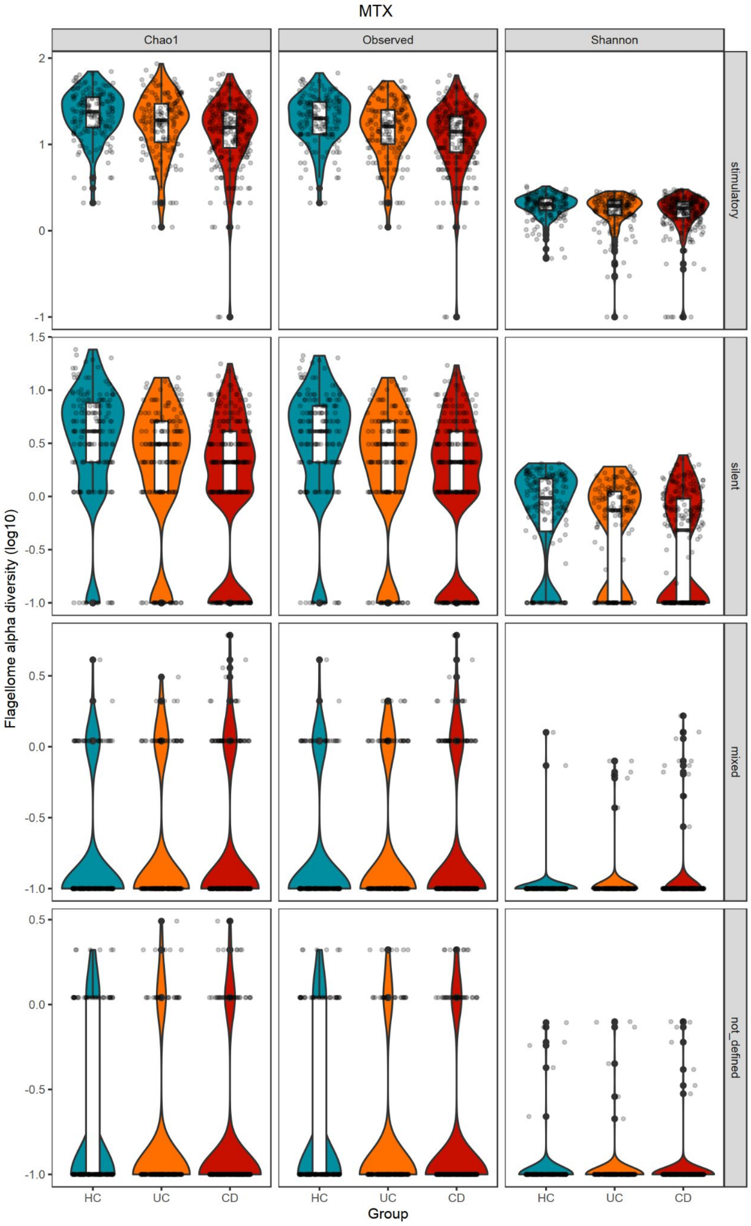

MTX
Chao1
Observed
Shannon
stimulatory
silent
mixed
not_defined
Flagellome alpha diversity (log10)
HC
UC
CD
Group

**SFigure 8**

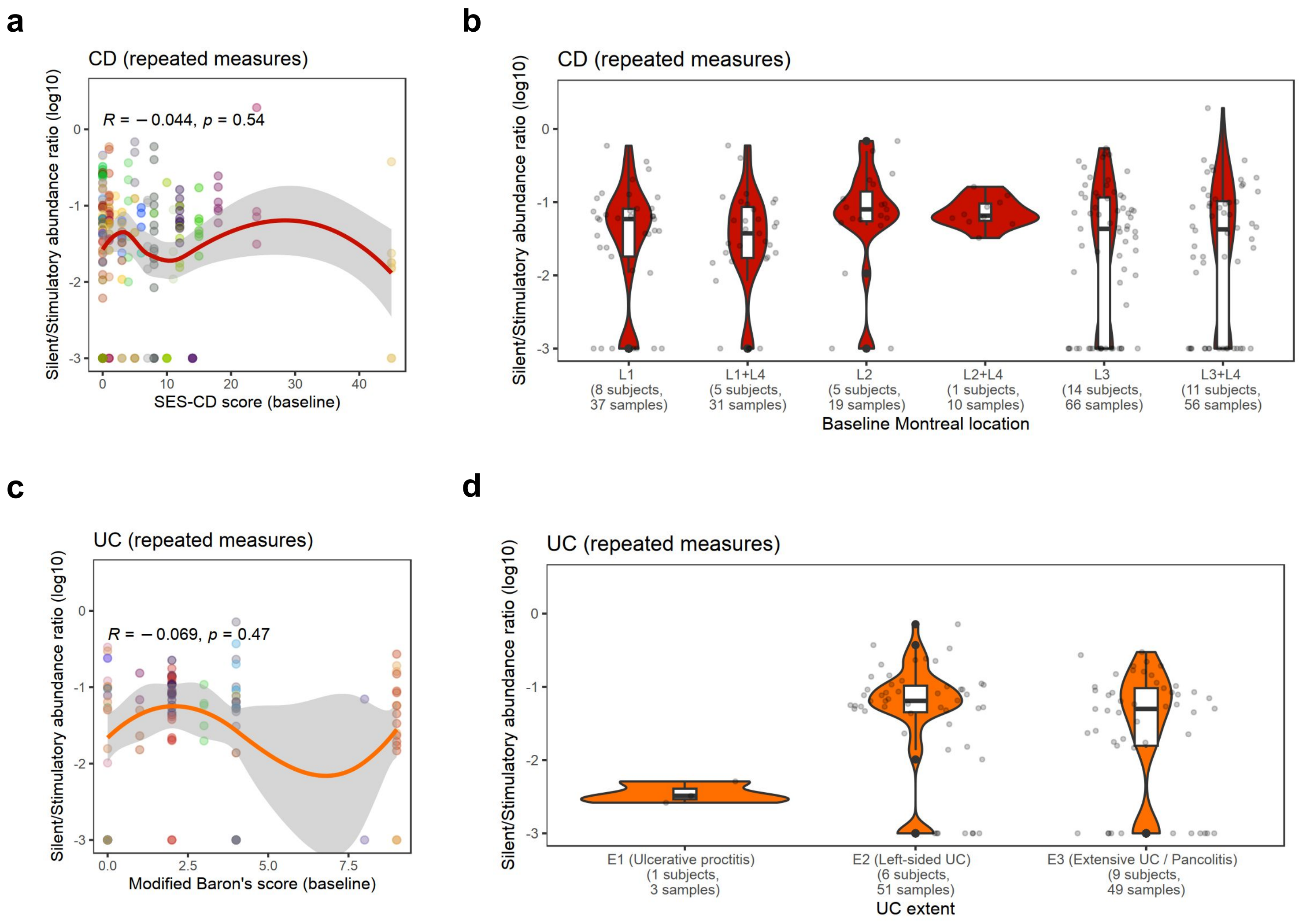
a
CD (repeated measures)
R = −0.044, p = 0.54
Silent/Stimulatory abundance ratio (log10)
SES-CD score (baseline)
b
CD (repeated measures)
Silent/Stimulatory abundance ratio (log10)
L1 (8 subjects, 37 samples)
L1+L4 (5 subjects, 31 samples)
L2 (5 subjects, 19 samples)
L2+L4 (1 subjects, 10 samples)
L3 (14 subjects, 66 samples)
L3+L4 (11 subjects, 56 samples)
Baseline Montreal location
c
UC (repeated measures)
R = −0.069, p = 0.47
Silent/Stimulatory abundance ratio (log10)
Modified Baron's score (baseline)
d
UC (repeated measures)
Silent/Stimulatory abundance ratio (log10)
E1 (Ulcerative proctitis) (1 subjects, 3 samples)
E2 (Left-sided UC) (6 subjects, 51 samples)
E3 (Extensive UC / Pancolitis) (9 subjects, 49 samples)
UC extent

# SFigure 9

**a**

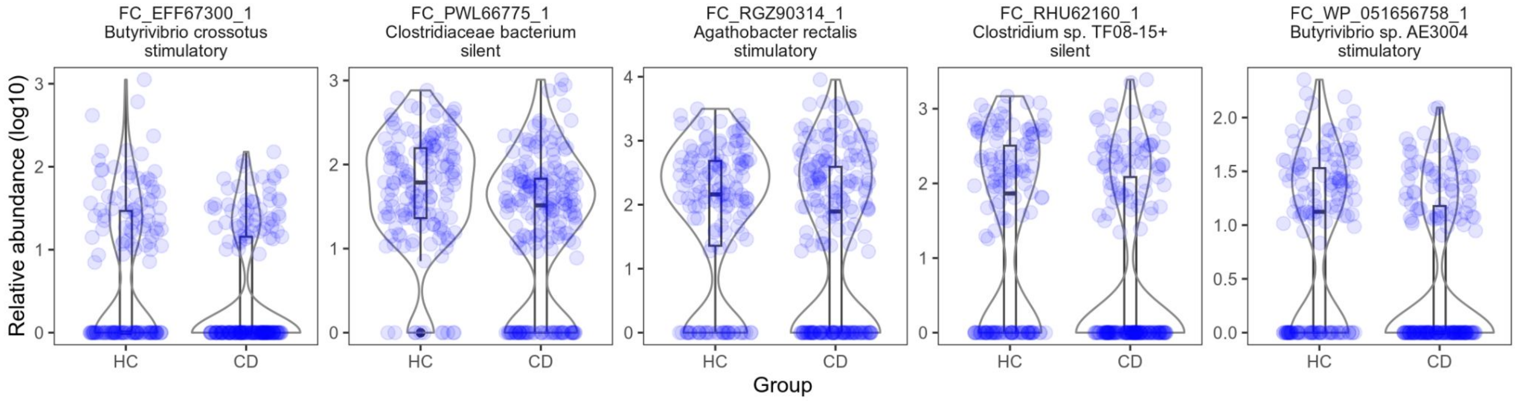


**b**

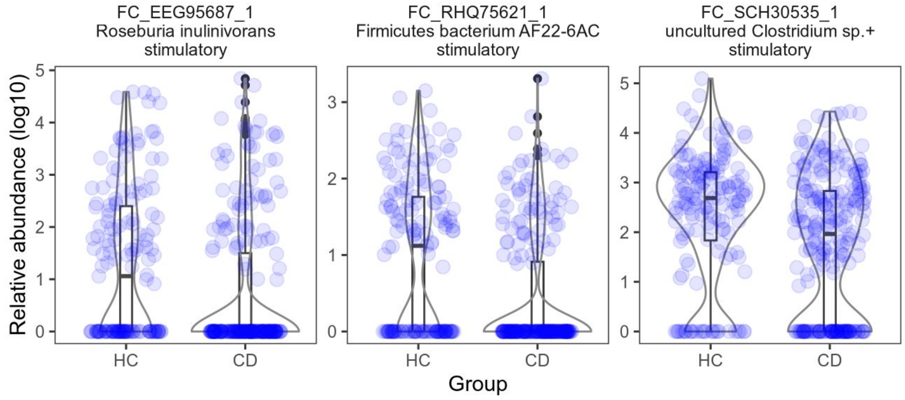


**c**

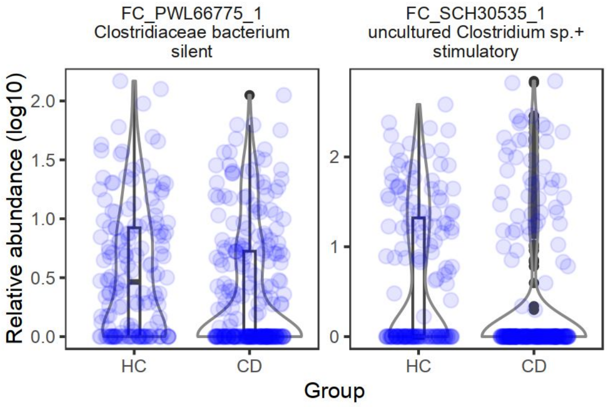

Supplement: Suppl_Figures.pdf — Supplemental Material [file KGMI_A_2698917_SM4689.pdf]
